# Chronic harmine treatment has a delayed effect on mobility in control and socially defeated rats

**DOI:** 10.1007/s00213-020-05483-2

**Published:** 2020-02-22

**Authors:** Bruno Lima Giacobbo, Janine Doorduin, Rodrigo Moraga-Amaro, Luiza Reali Nazario, Anna Schildt, Elke Bromberg, Rudi A.J.O. Dierckx, Erik F.J. de Vries

**Affiliations:** 1 Department of Nuclear Medicine and Molecular Imaging, University of Groningen, University Medical Center Groningen, Hanzeplein 1, 9713 GZ Groningen, the Netherlands; 2 Laboratory of Biology and Nervous System Development, Pontifícia Universidade Católica do Rio Grande do Sul, Porto Alegre, Brazil; 3 Laboratory of Neurochemistry and Psychopharmacology, Pontifícia Universidade Católica do Rio Grande do Sul, Porto Alegre, Brazil; 4 National Institute of Science and Technology for Translational Medicine (INCT-TM), Conselho Nacional de Desenvolvimento Cientifico e Tecnologico (CNPq), Brasília, Brazil

**Keywords:** Major depressive disorder, Neuroinflammation, Harmine, Monoamine oxidase inhibitors, Behavior, PET imaging

## Abstract

**Introduction:**

Depression is characterized by behavioral, cognitive and physiological changes, imposing a major burden on the overall wellbeing of the patient. Some evidence indicates that social stress, changes in growth factors (e.g., brain-derived neurotrophic factor (BDNF)), and neuroinflammation are involved in the development and progression of the disease. The monoamine oxidase A inhibitor drug harmine was suggested to have both antidepressant and anti-inflammatory properties and may, therefore, be a potential candidate for treatment of depression.

**Aim:**

The goal of this study was to assess the effects of harmine on behavior, brain BDNF levels, and microglia activation in control rats and a rat model of social stress.

**Material and methods:**

Rats were submitted to 5 consecutive days of repeated social defeat (RSD) or control conditions. Animals were treated daily with harmine (15 mg/kg) or vehicle from day 3 until the end of the experiment. To assess the effects of harmine treatment on behavior, the sucrose preference test (SPT) was performed on days 1, 6, and 15, the open field test (OFT) on days 6 and 14, and the novel object recognition test (NOR) on day 16. Brain microgliosis was assessed using [^11^C]PBR-28 PET on day 17. Animals were terminated on day 17, and BDNF protein concentrations in the hippocampus and frontal cortex were analyzed using ELISA.

**Results:**

RSD significantly decreased bodyweight and increased anxiety and anhedonia-related parameters in the OFT and SPT on day 6, but these behavioral effects were not observed anymore on day 14/15. Harmine treatment caused a significant reduction in bodyweight gain in both groups, induced anhedonia in the SPT on day 6, and significantly reduced the mobility and exploratory behavior of the animals in the OFT mainly on day 14. PET imaging and the NOR test did not show any significant effects on microglia activation and memory, respectively. BDNF protein concentrations in the hippocampus and frontal cortex were not significantly affected by either RSD or harmine treatment.

**Discussion:**

Harmine was not able to reverse the acute effects of RSD on anxiety and anhedonia and even aggravated the effect of RSD on bodyweight loss. Moreover, harmine treatment caused unexpected side effects on general locomotion, both in RSD and control animals, but did not influence glial activation status and BDNF concentrations in the brain. In this model, RSD-induced stress was not strong enough to induce long-term effects on the behavior, neuroinflammation, or BDNF protein concentration. Thus, the efficacy of harmine treatment on these delayed parameters needs to be further evaluated in more severe models of chronic stress.

## Introduction

Major depressive disorder (MDD) is a psychiatric disease that affects the daily life of millions of people and poses a burden to healthcare systems worldwide (Whiteford et al. 2013). Depression is mainly characterized by the loss of willingness to perform activities, sleeping and eating problems, sadness, and social isolation. Clinical and preclinical research indicates that decreased neurotransmitter and growth factor activation, microgliosis, and astrocytosis are involved in the pathogenesis of depression (Yirmiya et al. 2015; McKlveen et al. 2016; Kim and Na 2016). Neuroinflammation was suggested to play a major role in stress response to internal and external challenges, and increased inflammatory markers have been reported in MDD patients (Setiawan et al. 2015; Furtado and Katzman 2015), leading to the hypothesis of neuroinflammation-derived depression. Although the mechanisms are not completely understood, it is possible that brain inflammation may be caused by severe or prolonged stressful events and in turn cause some of the symptoms associated with MDD.

At the same time, drugs that bind to new targets are needed, and new therapeutic agents that bind to already clinically established targets are also needed, especially those that can act in more than one way or control more than one symptom. Monoamine oxidase-B (MAO-B) inhibitors have been used as treatment for MDD and other mood disorders for a long time. However, monoamine oxidase-A (MAO-A) inhibitors may also be used for this purpose, as the pathways of both enzymes are intimately related in the metabolism of monoamines. In the brain, the main function of MAO-A is the degradation of neurotransmitters, such as serotonin (5-HT), dopamine and norepinephrine, and blocking their release into the synaptic cleft (Youdim et al. 2006; Jiang et al. 2019). Like many other interventions used for depression, however, there is a large variability of treatment efficacy of MAO-A inhibitors, with a large percentage of MDD patients showing partial or no remission of symptomatology (Sinyor et al. 2010). Harmine is a β-carboline alkaloid derived from *B. caapi* (Malpighiaceae) found mainly in the Amazon rainforest of South America. Its main mechanism of action is through reversible inhibition of MAO-A (Iurlo et al. 2002). Harmine is metabolized in the liver into harmol and hydroxylated harmine and excreted both via the intestines and the kidneys (Zetler et al. 1974; Jiang et al. 2015; Zhao et al. 2012). Harmine may be an interesting candidate drug as it shows not only antidepressant (Fortunato et al. 2009; Réus et al. 2010b; Liu et al. 2017a) but also anti-inflammatory properties (Liu et al. 2017b; Li et al. 2018).

Although harmine can be an interesting candidate for the treatment of depression and anxiety, its effects on the organism are not yet clear; thus, turning this compound into a prospective anti-depressant still requires a significant amount of steps. The goal of this study is to assess the short-term and delayed effects of a daily dose of harmine on behavior of normal rats and rats submitted to a protocol of psychosocial stress, i.e., repeated social defeat (RSD). RSD is considered a model of MDD for its ability to emulate psychosocial stressors of human depression in an animal model by using territoriality and hierarchical status as motivators. The effects of harmine treatment on anhedonia, explorative behavior, anxiety, and memory were measured with the sucrose preference test (SPT), the open field test (OFT), and the novel object recognition test (NOR), respectively. [^11^C]PBR28 PET of the brain was performed to assess the delayed effect of harmine on stress-induced neuroinflammation in various brain regions. In addition, frontal cortex and hippocampus were collected after termination for BDNF protein concentration analysis. BDNF is a protein associated with neuronal integrity and neuroprotection.

## Material and methods

### Animals and drug

The study protocol complied to European Directive 2010/63/EU and the Law on Animal Experiments of The Netherlands; it was approved by the Central Committee on Animal Experiments of The Netherlands (The Hague, license no. AVD1050020171706) and the Institutional Animal Care and Use Committee of the University of Groningen (IvD 171,706–01-006). Male Wistar rats (HsdCpb:WU, 8 weeks old – Envigo, The Netherlands) were housed individually at the Central Animal Facility of the University Medical Center Groningen. Prior to the experiments, animals were habituated to the facility for at least 7 days. Animals were maintained in rooms with controlled temperature (21 ± 2 °C) and humidity in a 12/12 h light/dark cycle (lights off at 08:00 P.M.), with food and water provided ad libitum. After acclimatization, animals were randomly divided into 4 groups (8 animals per group) according to harmine treatment (harmine or vehicle) and social defeat protocol (RSD or control). Harmine hydrochloride (Santa Cruz biotechnology; sc-295136B) was diluted in saline to the desired concentration of 15 mg/kg in a volume of 1 ml. The solution was then heated up to 50 °C and stirred with ultrasound until it became a clear solution (about 10 min). When injected in the animals, the solution was at room temperature.

### Study design

**Fig. 1:**
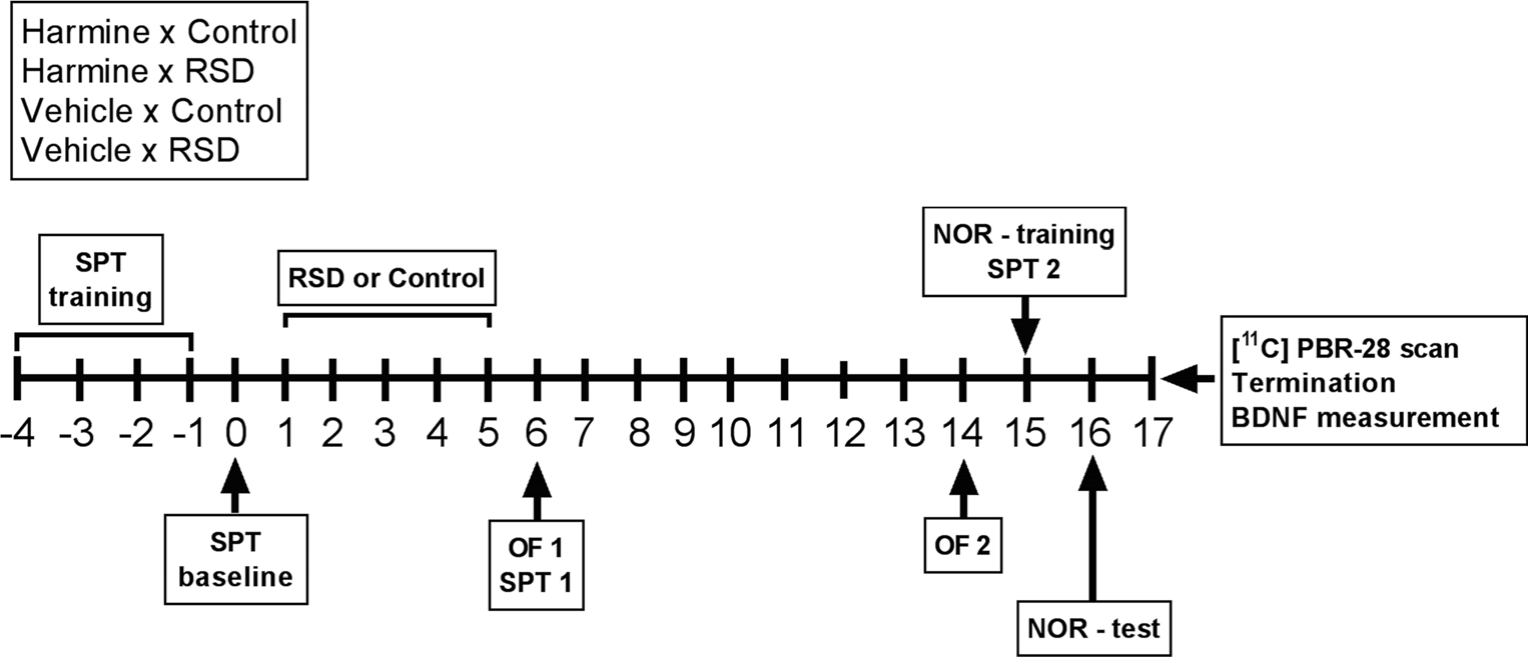
Design of the study. Abbreviations: SPT, sucrose preference test; RSD, repeated social defeat; OF, open field test; NOR, novel object recognition

A summary of the experiment is presented in Fig. 1. Five days before the beginning of RSD, the animals were daily trained for the SPT for 1 h (SPT – training). The first SPT (day 0) was performed during the night before the first day of RSD. Animals were then submitted to the RSD protocol daily for 5 days. The second SPT and the first OFT were performed one day after the last RSD trial (day 6). On the third day of RSD, harmine or vehicle administration was started, which lasted until the end of the experimental phase (day 3–17). Nine days after RSD (day 14), animals were submitted to the second OFT. On day 15, the third SPT test and the training for the NOR test were performed. On day 16, the NOR test was done. On day 17, a [^11^C]PBR28 PET scan was acquired before termination of the animals and collection of brain tissue for further analysis. Animals were weighed daily from day 1 to 17, always before drug administration.

### Drug administration

From day 3 to 17, defeated and control rats were submitted to a daily intraperitoneal injection of either harmine (15 mg/kg) or vehicle solution. Harmine caused slight tremors 1 min after injection, as was previously described in the literature (Cox and Potkonjak 1971). In our study, the effect lasted for 45–60 min, and the behavior of the animals returned to normal after this period. To avoid acute effects of harmine on the RSD protocol or behavioral parameters, RSD and behavioral assessment were performed in the morning (09:00–12:00 A.M.), while harmine administration was performed in the afternoon (13:00–14:00 P.M.).

### Repeated social defeat

Before the social defeat protocol, 12-weeks old male Long-Evans rats (residents, HsdBlu:LE, Harlan, The Netherlands; *n* = 6; weight, 450–500 g at the beginning of RSD protocol) were housed with females of the same age in a large wooden cage (80 × 50 × 40 cm) with a plastic lid. This setup allowed the resident to develop territorial behavior over a large area. The Long-Evans rats were submitted to a training social defeat protocol to allow for the selection of their aggressiveness prior to the beginning of the first RSD protocol. Animals that showed an attack latency (i.e., time to initiate the first attack) of 60 s or less and no signs of violent behavior (i.e., attack latency of less than 3 s without threatening behavior before the first attack) during the 5 days of training were selected for the study. Long-Evans rats that showed non-aggressive or over-aggressive behavior were excluded from the study.

One hour before the beginning of RSD, the female rat was removed from the resident’s cage. Then an experimental animal (intruder, Wistar rat) was placed in the resident’s cage to begin the defeat protocol. Attack latency and submission time (i.e., time the intruder takes to show a submissive posture for at least 3 s) were measured. After the intruder displayed a submissive posture, it was placed in a wire mesh cage (40 × 20 × 20 cm) inside the resident’s cage for 60 min. By placing the intruder in a wire mesh, there is no physical contact between intruder and resident anymore, but the intruder is still aware of the presence of the aggressive resident. After 60 min, the intruder is removed from the resident’s cage and placed back to its home cage, and the female is placed back in the resident’s cage. Control animals were placed in a large, plastic cage for 10 min without resident and subsequently put in the wire mesh cage for 60 min. Then the animals were placed back to their home cages. This protocol was repeated on five consecutive days, and the intruder was always introduced to a different resident.

### Open field test (OFT)

OFT were performed on day 6 and 14 to observe the acute and delayed effects of harmine treatment in control and RSD animals. To avoid habituation effects, two different arenas were used for the trials. For the first OFT, a round wooden arena of 80-cm diameter was used, whereas a square arena of 50 × 50 cm^2^ was used for the second trial. For both tests, the animal was placed in the room 1 h before the experiment and left alone during this period. After 1 h, the investigator placed the animal in the arena facing the wall and started recording its exploratory behavior for 6 min, after which the animal was placed back into its home cage. The arena was cleaned with ethanol 70% and dried with paper tissues after each test. Analysis of the total distance and the time the animal moved, its velocity, and the time spent in the center and in the periphery of the arena was performed using Ethovision XT 14.0 software (Noldus, The Netherlands). The number of times the animal explored the environment (rearing), the number of times the animal spent grooming, and the time the animal spent immobile (freezing) were measured manually by the investigator.

### Novel object recognition test (NOR)

The NOR test was performed in the circular OFT arena. The test was performed on day 15 (training) and 16 (long-term memory). For training, two identical objects (A and A’ – plastic cylinders) were placed 20 cm from the wall and 20 cm from the center on opposite sides of the field. Thus, the animal had plenty of space to explore the environment and interact with the objects separately. For training, the animals were placed in the arena and allowed to explore the objects. When the animal had explored each object for 30 s, the training protocol was ended, and the animal was returned to its cage. If the animal did not reach the exploration criteria after 8 min, the training protocol was also ended, and the animal was returned to its cage.

For the long-term memory test, the animals are placed back into the arena 24 h after training, but with one object being replaced by an object with a different shape and color (A’ replaced by B – piled Lego bricks). The animal was placed near the wall of the arena facing the objects and left to explore freely. During the whole test, the animal was recorded. After 6 min, the animal was retrieved and placed back in its home cage. After each trial, the objects and apparatus are cleaned with 70% ethanol and dried with paper tissues. Analysis of the time spent exploring objects A and B were analyzed automatically with Ethovision XT 14.0. The recognition index (RI) was defined as the time spent exploring object B divided by the total amount of time exploring both A and B. Animals that explored the objects for less than 5 s were excluded from data analysis.

### Sucrose preference test (SPT)

Animals were habituated to the SPT protocol by replacing their water bottle for a bottle containing 1% sucrose for 1 h on 4 consecutive days. For the test SPT, 2 identical bottles – one containing drinking water and the other containing 1% sucrose solution – were placed in the cage of the rat and left overnight (placement of bottles at 03:00–04:00 P.M.). The next day, the bottles were removed (at 10:00 A.M.) and weighed to estimate the amount of fluid consumed by the animal. The percentage of sucrose intake was calculated from the weight difference of the 1% sucrose bottle divided by the sum of the weight differences of both bottles.

### Positron emission tomography (PET)

[^11^C]PBR28 PET was performed on small animal PET scanner (Focus 220, Siemens Medical Solutions, USA) with constant monitoring of the animal’s heart rate and blood oxygen levels. Anesthesia was induced with 5% isoflurane and maintained with 2% isoflurane. After anesthesia induction, a cannula was inserted in the lateral tail vein for tracer injection. [^11^C]PBR28 (49.6 ± 3.3 MBq) was injected as a bolus, and the animal was placed in its home cage for 30 min. Then the animal was anesthetized again and a transmission scan with a Co-57 source was performed for the correction of attenuation and scatter. A 30-min emission scan was started 45 min after tracer injection.

Images were iteratively reconstructed (OSEM2D, 4 iterations and 16 subsets) after correction for attenuation and radioactive decay. The reconstructed PET images were automatically co-registered to a [^11^C]PBR28 rat brain template using PMOD software (PMOD Technologies LLC, Switzerland). Regions of interest (ROIs) were delineated for the following regions: amygdala, cerebellum, corpus callosum, midbrain, frontal cortex, temporal cortex, dorsal cortex, hippocampus, hypothalamus, brainstem, olfactory nucleus, thalamus, and striatum. The average uptake in the ROI’s (in kBq/cc) was corrected for the injected tracer dose and the bodyweight of the animals and expressed as standardized uptake value (SUV).

### BDNF analysis

After the PET scan, the animals were transcardially perfused with cold phosphate-buffered saline pH 7.4 (PBS), and the brain was removed for tissue analysis. The frontal cortex and hippocampus were excised from the brain, placed in an ice-cold PBS solution, snap-frozen in liquid nitrogen, and stored at − 80 °C until further analysis. RIPA buffer (Sigma-Aldrich, R0278 – containing 150 mM NaCl, 1.0% IGEPAL® CA-630, 0.5% sodium deoxycholate, 0.1% SDS, 50 mM Tris, pH 8.0) was added to the brain tissue (50-μl/mg tissue) and cooled on ice. The tissue was pounded until no solid fragments were visible anymore. The homogenized tissue was centrifuged at 12,000 rpm for 15 min. The supernatant was collected for total protein quantification by the bicinchoninic acid assay (BCA) using bovine serum albumin as a standard. Then, BDNF was measured with ELISA (Cloud-clone, SEA011Ra) according to the manufacturer instructions. Intra-assay precision was < 10%. Tissue lysate was diluted 1:5 in PBS (five samples were diluted 1:6 due to low amount of lysate). Samples were read at 450 nm and corrected for the total amount of protein.

### Statistical analysis

Statistical analyses were performed using the two-way generalized linear model (GLM) with RSD and harmine treatment as factors. A within-subject factor (time) was added to the SPT analysis. The main effects of RSD and harmine were evaluated, as well as the interaction between both factors and time, whenever needed. For all tests, *p* < 0.05 was considered statistically significant. SPSS 23 (IBM, United States) was used for all statistical analyses.

## Results

### Social defeat and harmine treatment decrease bodyweight gain

**Fig. 2:**
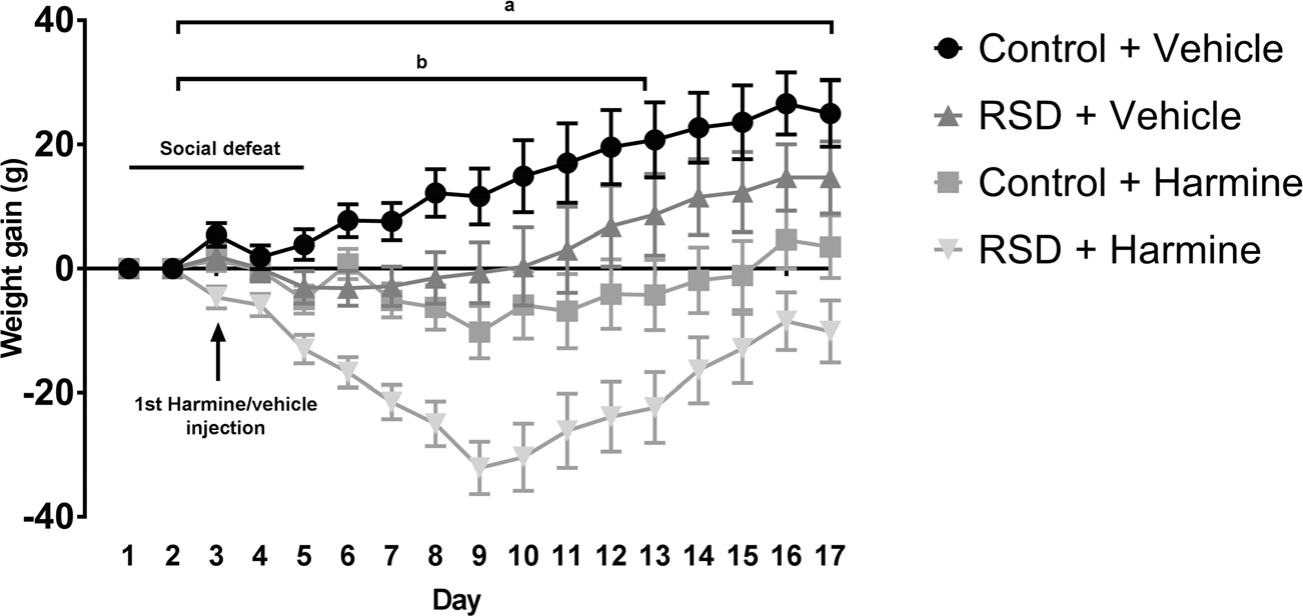
Bodyweight change over time. **a***p* < 0.05 between harmine and vehicle treatment for each time point until day 17. **b***p* < 0.05 between RSD and control for each time point until day 12. Points and whiskers represent mean ± SEM

**Fig. 3:**
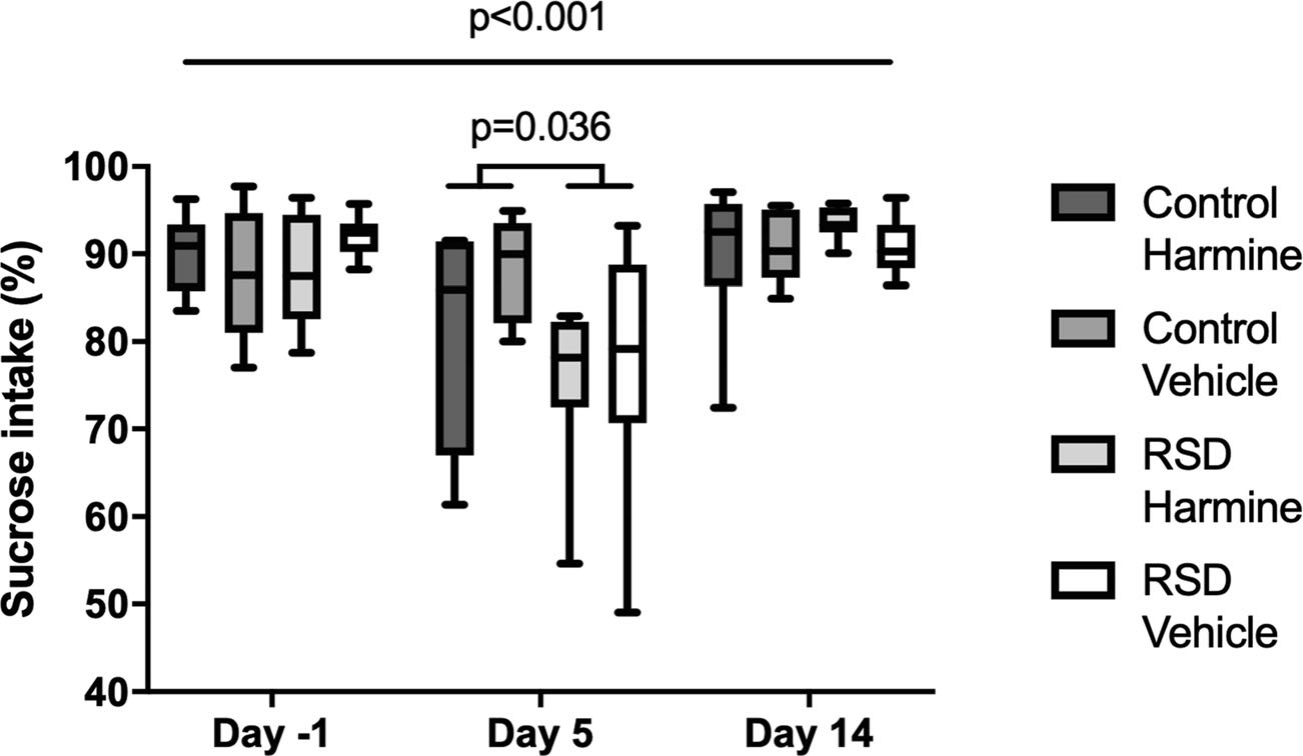
Sucrose preference on day 1, 5, and 14 showing the effects of RSD and harmine treatment. The horizontal line and whiskers represent median ± 95% CI, respectively. A main effect of time was observed (*p* < 0.001). In addition, a significant effect of RSD on sucrose preference was found on day 5 (*p* < 0.05)

Figure 2 depicts the effect of RSD and harmine on bodyweight gain over time. As expected, there was a significant effect of time on bodyweight within animals (*F* = 11.380, *p* < 0.001). Additionally, there was a significant effect of RSD and harmine treatment on bodyweight gain (RSD, *F* = 3.275; *p* = 0.040; Harmine, *F* = 0.192; *p* < 0.001), but no interaction between RSD and harmine treatment (*p* > 0.05). RSD induced a significant reduction in bodyweight gain compared to the control groups (F_(1,25)_ = 12.123, *p* = 0.002), and also, harmine treatment caused a significant reduction in bodyweight gain when compared to vehicle treated controls (*F*_(1.25)_ = 28.624, *p* < 0.001) Figure 3.

Harmine treatment seemed to have a stronger effect on bodyweight than RSD, as harmine-treated animals showed a significant difference when compared to vehicle-treated animals until the end of the experiment (day 17 – *p* < 0.05 at all time points), with no return to normal levels when compared with vehicle animals. The significant reduction in bodyweight induced by RSD lasted only until day 12 (*p* < 0.05), after which the effect of RSD was normalized.

### Acute, but no long-term effect of harmine and RSD on sucrose intake

Sucrose preference test was performed on days 1, 6, and 15. There was a main effect of time (*F*_(2,54)_ = 17.270, *p* < 0.001) and a main effect of RSD (*F* = 3.797, *p* = 0.036) on SPT, but no main effect of harmine treatment or any interaction. Further pairwise comparisons showed a significant decrease in sucrose intake due to RSD on day 6, when compared with the other time points (RSD day 6 vs RSD days 1 and 15, *p* < 0.001), while such a temporal effect was not observed in controls.

Interestingly, pairwise comparisons within vehicle and harmine groups showed similar results as those found in control and RSD groups, with sucrose intake by harmine-treated animals being significantly lower on day 6 than on the other days (harmine day 6 vs harmine days 1 and 15, *p* < 0.001), but no difference between time points was observed in the vehicle groups.

### Transient effect of RSD on anxiety-like behavior

**Fig. 4:**
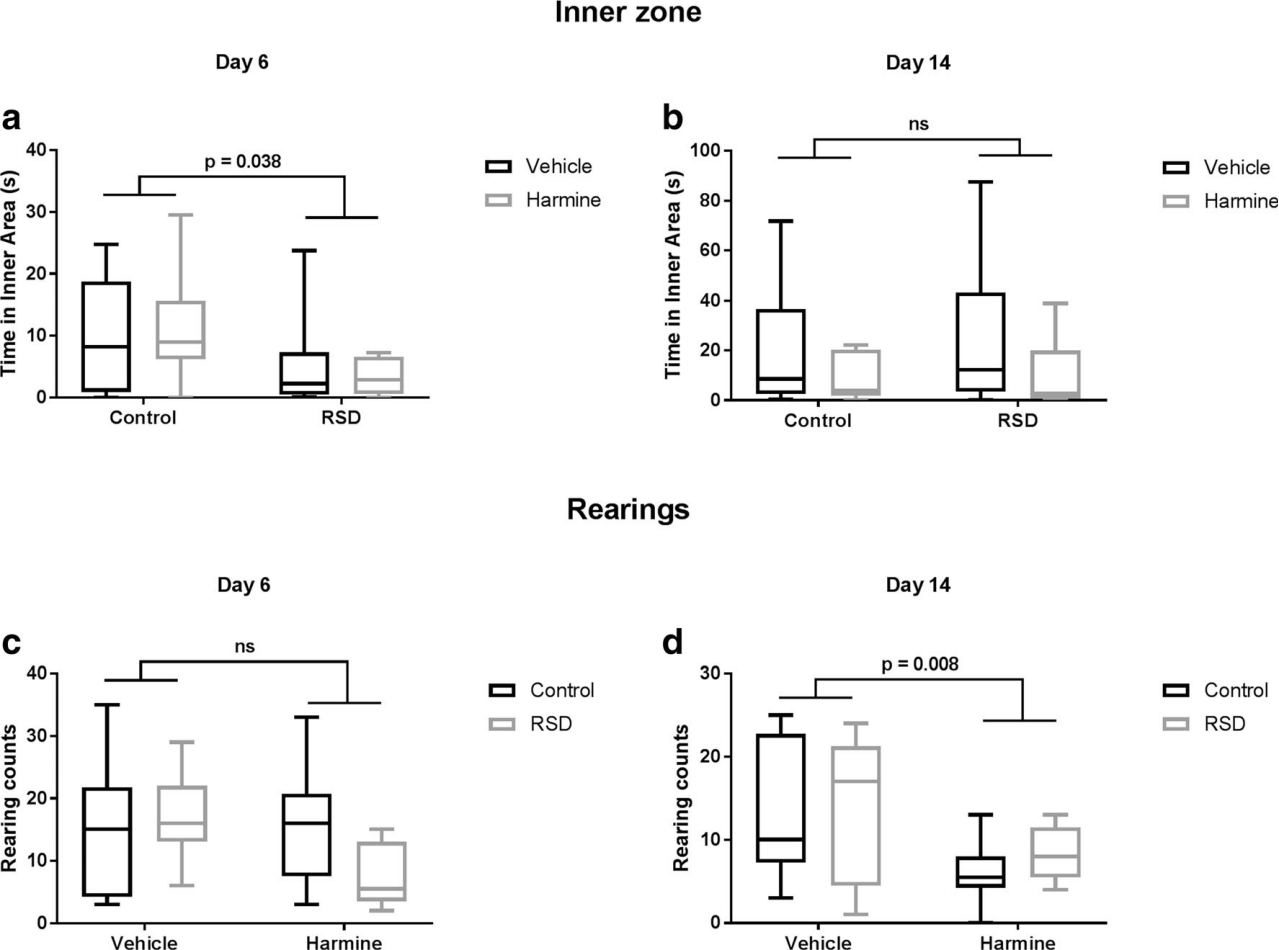
Effect of social defeat on the time spent in the inner zone of the arena (**a–b**) and number of rearings (**c–d**) in the OFT on day 6 (left panel) and day 14 (right panel). Horizontal lines and whiskers indicate median ± 95% CI, respectively; sample size, 7–8 animals per group

The effect of RSD and harmine treatment on anxiety was assessed by the time the animal spent in the center of the arena during the OFT. As expected, animals that underwent RSD spent significantly less time in the center of the arena on day 6 (*F* = 4.747, *p* = 0.038 – Fig. 4). However, no difference between animals injected with harmine and vehicle was found. There were no significant interactions between RSD and harmine treatment, suggesting that harmine was unable to reverse the effects of RSD. On day 14, animals submitted to an RSD protocol did not show any anxiety-like behavior anymore, as shown by the lack of significance between RSD and controls. Treatment with harmine had no long-term effect on the anxiety-like behavior either(all *p* > 0.05).

### Effect of harmine on mobility

**Fig. 5:**
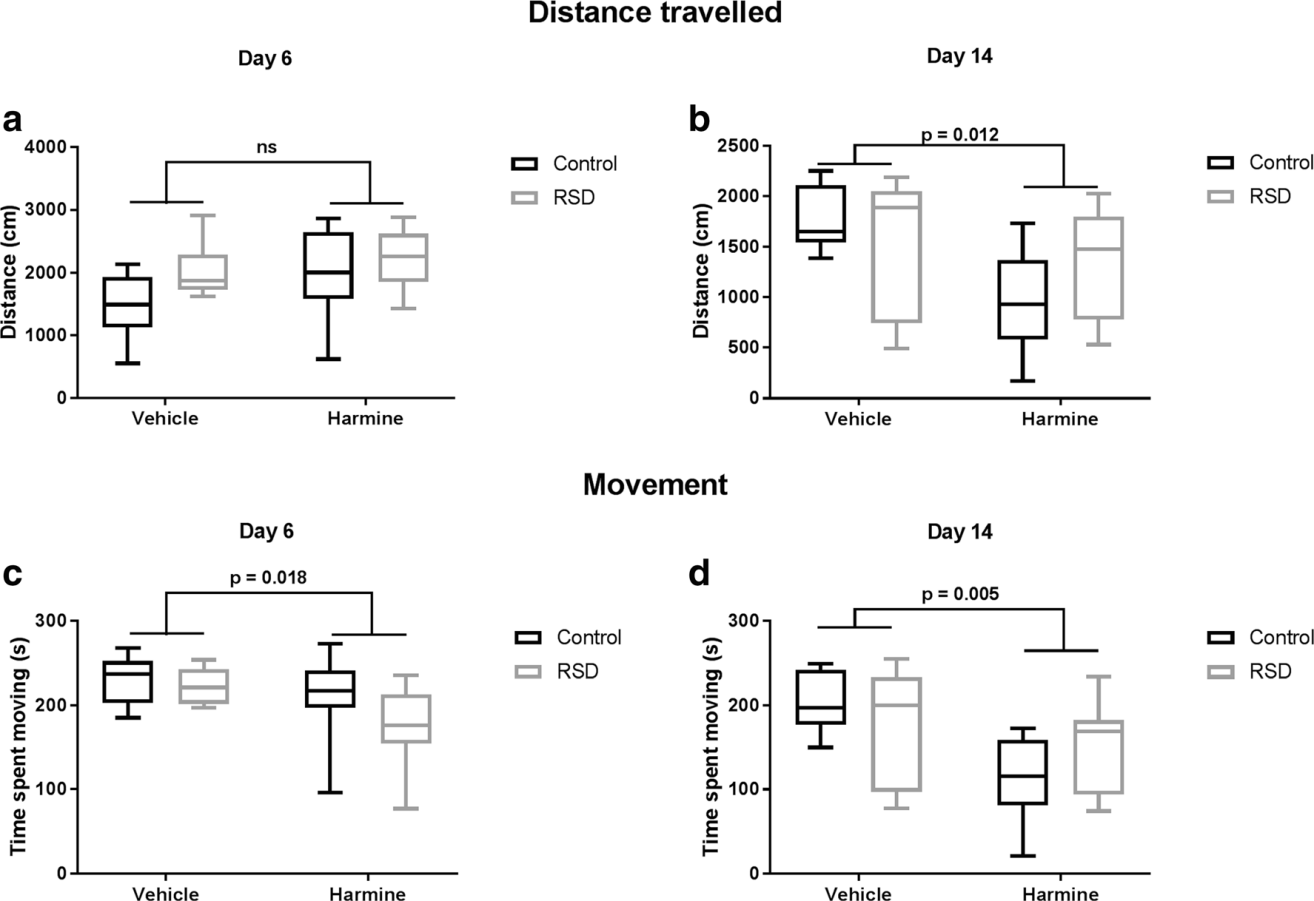
Acute (left panel) and long-term (right panel) effect of harmine treatment on the distance traveled (**a–b**) and time spent on movement (**c–d**). Horizontal lines and whiskers indicate median ± 95% CI, respectively; sample size, 7–8 animals per group

Harmine treatment significantly reduced the time the animal spent moving in the OFT on day 6 (*F* = 6.356, *p* = 0.018) and day 14 (*F* = 7.283, *p* = 0.012 – Fig. 5). Likewise, the total distance moved by harmine-treated animals was significantly smaller than the distance traveled by vehicle-treated animals (*F* = 7.283, *p* = 0.012). RSD did not have any effect on mobility neither on day 6 nor on day 14.

Additionally, there was a significant effect of harmine treatment on rearing (exploratory behavior). Animals administered with harmine display less frequently a rearing posture (*F* = 4.475, *p* = 0.012). RSD did not have any effect on rearing frequency (Fig. 4).

### No effect of harmine treatment on long-term memory

**Fig. 6:**
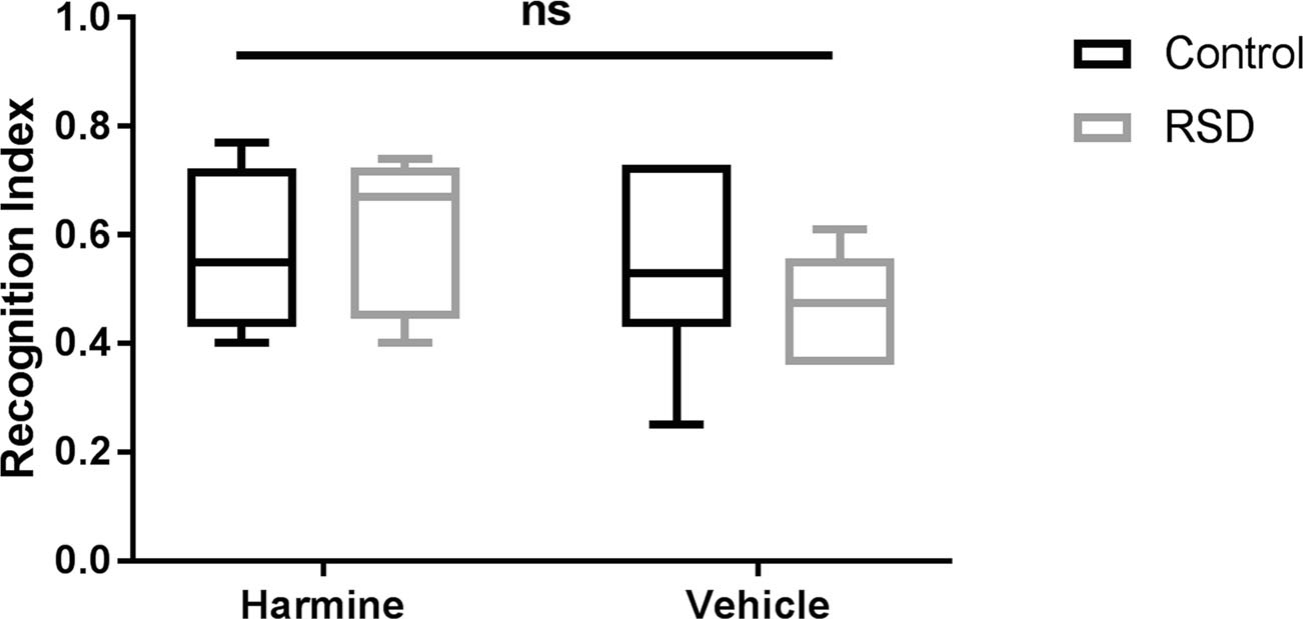
Results of the NOR test on day 16, showing no effect of RSD or harmine treatment on long-term memory. Horizontal lines and whiskers indicate median ± 95% CI, respectively; sample size, 6–7 animals per group

The NOR test did not show any effect of harmine or RSD on long-term memory, as no significant differences in recognition index between groups were observed (*p* > 0.05) (Fig. 6).

### No effect of harmine treatment on glial activation

**Fig. 7:**
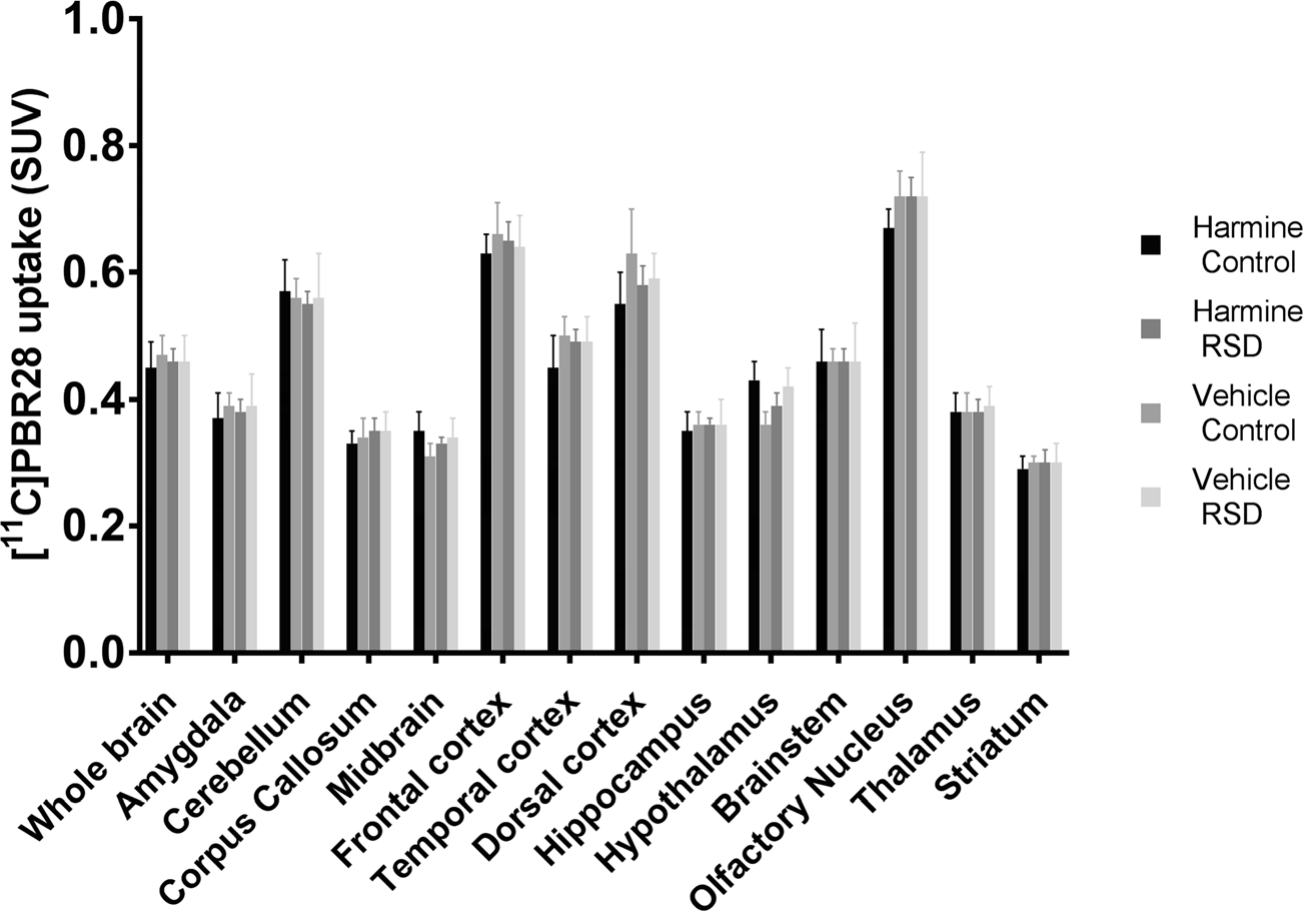
Results of [11C]PBR28 PET, showing no significant effect of RSD or harmine treatment on tracer uptake (SUV) in any brain region of interest. Bars and error bars represent mean and SEM, respectively; sample size, 7–8 animals per group

For all groups, [^11^C]PBR28 PET showed highest tracer uptake in the olfactory nucleus, frontal and dorsal cortex, and cerebellum. However, stress-induced glial activation could not be detected on day 17, as there was no significant effect of RSD on the uptake (SUV) of [^11^C]PBR28 in any of the brain regions assessed (all *p* > 0.05 – Fig. 7). Harmine did not have any effect on the glial activation status either, as [^11^C]PBR28 PET did not show any significant differences, neither in the control group nor in the RSD group.

### No chronic effect of harmine treatment on BDNF concentration

**Fig. 8:**
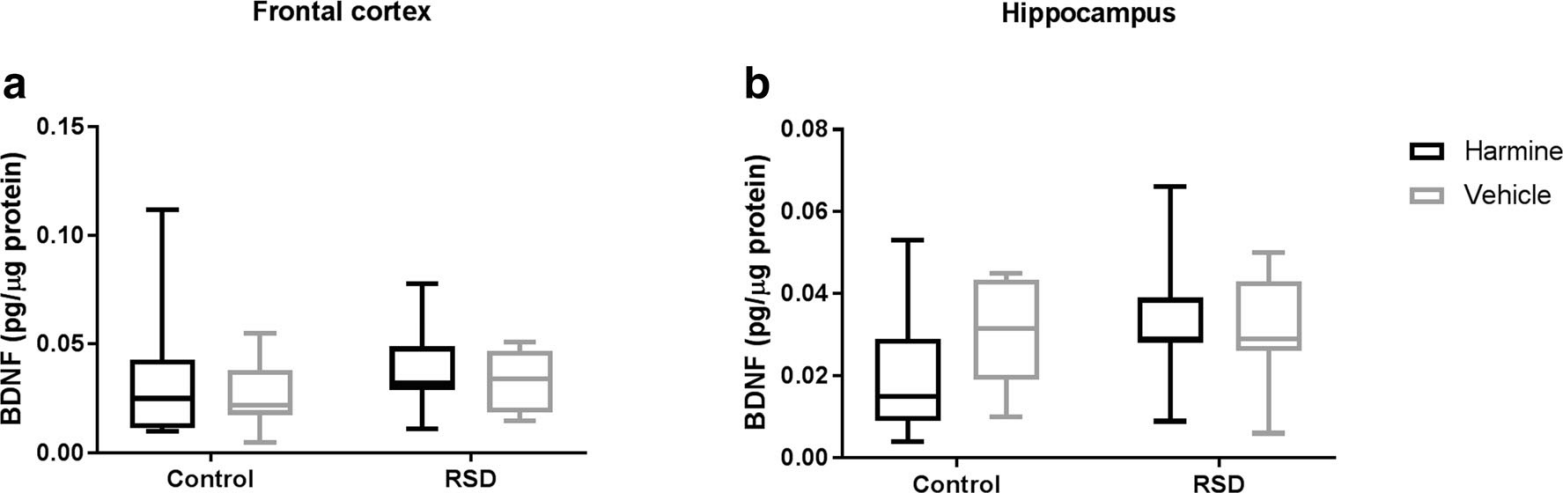
BDNF concentration in hippocampus and frontal cortex on day 17, showing no significant effects of RSD or harmine treatment. BDNF concentrations are corrected for the total concentration of protein. Horizontal lines and error bars represent mean ± 95% CI, respectively; sample size, 5–8 samples per group

There was no significant main effect of treatment with harmine or RSD on the BDNF concentration in the hippocampus or frontal cortex (*p* > 0.05 – Fig. 8). As BDNF is highly correlated with cognitive parameters, we also assessed if there was a significant relationship between memory and the concentration of BDNF in either brain region using linear regression, but did not find any correlation.

## Discussion

This study aimed to assess the effect of harmine on behavior, glial activation, and BDNF concentrations, both in healthy rats and rats submitted to an RSD protocol. Harmine proved unable to reverse the acute anxiety and anhedonia induced by the RSD protocol. However, harmine had a negative long-term effect on the bodyweight gain, especially in rats exposed to a stressful situation. Additionally, harmine had a significant effect on the general locomotion, both on day 6 (3 days after first injection) and on day 14 (11 days after first injection), but did not affect long-term memory, glial activation status, or BDNF concentrations on day 16/17.

### Harmine is unable to reverse RSD -induced anxiety and depressive-like behavior

Animals submitted to RSD showed more anxiety (time spent in the center of the open field arena) and depressive-like behavior (preference of sucrose solution over water) than controls. These results are supported by literature showing that several stressors can induce anxiety and depression-associated parameters in animals (Riga et al. 2015; Liu et al. 2017a, c) (Miczek et al. 2008; Wohleb et al. 2014). Although our results show an acute increase of anxiety and depression-associated measures in animals submitted to RSD, this effect did not last until 9 days after RSD. This transient effect has previously been observed in various RSD protocols, using different species, number of defeats, RSD duration, and evaluation period. Kopschina Feltes and colleagues observed in Wistar rats that the effect of a similar RSD protocol was observed 1 week after RSD but was resolved after 90 days (Kopschina Feltes et al. 2019). Martin and colleagues used a modified 10-day RSD protocol on C57BL mice and found that the transient effect of RSD had normalized after 18 days (Martin et al. 2017). Gottschalk et al. found that a 5-day RSD protocol has a significant effect on anhedonia 3 days after the last RSD exposure (Gottschalk et al. 2018). We also reported a significant effect of RSD on anhedonic behavior, although the SPT showed a large variability 1 day after RSD. We hypothesize that this variance can be the effect of the resilience/proneness of the animals toward stress. Krishnan and colleagues also found a strong variance in a large cohort of mice submitted to social defeat stress, with a remarkable difference between resilient and prone animals toward stress (Krishnan et al. 2007). Febbraro and colleagues recently showed a similar difference in response in rats after chronic mild stress (Febbraro et al. 2017).

Both MAO-A and MAO-B inhibitors have historically been used as an antidepressant agent with variable effectiveness. Inhibition of either MOA subtype leads to reduced monoamine degradation and consequently increased monoamine levels in the synaptic cleft (Youdim et al. 2006; Finberg and Rabey 2016). Our study shows that acute administration of MAO-A inhibitor harmine was unable to improve the acute depressive-like state of the animals subjected to RSD. Although there are no studies on the acute therapeutic effect of harmine after social stress, it is known that mid- to long-term administration of harmine improves depressive-like symptomatology in animals submitted to a chronic unpredictable stress protocol (Abelaira et al. 2013). One study reported that chronic administration of harmine prior to application of chronic unpredictable stress was able to mitigate the stress-induced depressive-like behavior (Liu et al. 2017a). Another study investigated the therapeutic effect of chronic harmine administration for 1 week in chronically stressed rats and found similar results, using the preference for sugary food the main outcome measure (Fortunato et al. 2010). These studies, however, were performed in a chronic mild stress model (CMS) and therefore are difficult to translate to the stress protocol used in our study. When compared with CMS, RSD can be considered a more ecologically viable option for a stressor, as it uses the normal rodent behavior to present a stressful situation (i.e., fight-or-flight), thus improving its construct validity to what is observed in human stress studies. The lack of acute effect of harmine can be explained by its mechanism of action. In humans, MAO inhibitors are known to take two or more weeks for mitigation of depressive symptoms to occur. Therefore, short bouts of MAO inhibitors likely have no effect on symptomatology of depression. Another significant methodological difference between previous studies and our findings is the timing of drug administration. Other studies that assessed animal behavior did so 60 min after injection of the drug (Abelaira et al. 2013). It is plausible that the observations in previous studies were more related to acute (i.e., drug injection) rather than chronic effects of harmine administration. Our goal was to investigate the effect of chronic harmine treatment on the behavioral tasks and therefore we performed the behavioral tests before injection of the drug.

### Harmine and RSD cause bodyweight loss

Both RSD and harmine treatment caused a reduction in bodyweight. The effect of RSD is in line with previous literature describing that animals submitted to the RSD show transient weight loss – or a lower weight gain – during the period of such protocol (Becker et al. 2008; Kopschina Feltes et al. 2019). Stress can increase brown adipose tissue thermogenesis and hyperthermia and thus cause a reduction in bodyweight (Zhang and Bi 2015). The effect of harmine treatment on bodyweight might be due to a similar mechanism, as harmine is able to induce adipose tissue thermogenesis by blocking *Ucp1* gene inhibition by chromodomain helicase DNA binding protein 4 (CHD4) (Nie et al. 2016). Interestingly, the effect of harmine on bodyweight seemed to be exacerbated after exposure to a stressor, suggesting an interaction between harmine and stress mechanisms. One report has shown that the effect of harmine on adipocyte thermogenesis is related to activation of the ERK, p38, and AKT pathways (Nie et al. 2016). In the brain, these pathways are also involved in the modulation of several neuronal functions that may affect the stress response.

### Harmine reduces locomotion

Our findings show that harmine significantly decreased the general movement of the animals, as measured by their immobility time on day 6 and 14. Unlike our study, others have not observed any differences in locomotion after acute or chronic administration of harmine for 12 days (Fortunato et al. 2009; Réus et al. 2010a). However, another study showed that the harmine analogs, harmane, and norharmane induced a significant decrease in the distance traveled by the animals, but no differences in anxiety or motor coordination outcomes (Goodwin et al. 2015). Harmine has been suggested as a potential metabolite of harmane (Guan et al. 2001) and thus this metabolite, rather than harmane itself, could be responsible for the observed effect on locomotion. However, a recent study disputes harmine being a metabolite of harmane (Li et al. 2016), which would undermine this hypothesis.

Previous research has shown that acute administration of harmine causes tremorgenic effects on rats (Cox and Potkonjak 1971). In this study, we also found that harmine administration caused transient tremors, which lasted for approximately 60 min (data not shown). Although the tremors were not visible anymore after 1 h, it may have had some lingering effect on the general locomotion. Indeed, one of the main side effects of monoamine oxidase inhibitors is movement impairment due to increased serotonin neurotransmission (i.e., serotonin syndrome) (Brierley and Davidson 2012). Likewise, one could speculate that the reduced mobility induced by the administration of harmine in our study could be the cumulative effect of the daily treatment on serotonin neurotransmission. However, further investigation of the mechanisms for the effect of harmine on general locomotion is needed.

### RSD and harmine do not affect long-term memory, BDNF levels, or glial activation

In our study, RSD by itself was unable to induce long-term memory changes. This is similar to what was found previously in our laboratory (Kopschina Feltes et al. 2019). Other studies, however, showed that different subtypes of memory are affected by RSD. McKim and colleagues found that a 6 days RSD protocol was able to impair spatial memory recall, as assessed with the Morris and Barnes mazes (McKim et al. 2016). Wohleb and colleagues found that this effect lasts for up to 8 days, suggesting a subchronic effect of RSD (Wohleb et al. 2014). It is worth noting that memory tests can pose stressful environments and the NOR test is considered a substantially less stressful event than the Barnes maze or Morris water maze; comparison between the tests is therefore difficult.

A recent meta-analysis suggested that harmine is able to reverse stress-related memory impairment (dos Santos and Hallak 2017). However, this effect was not observed in our study, with both harmine and vehicle-treated animals showing similar memory performance whether a stressor was present or not. A similar study using chronic unpredictable stress (CUS) for 40 days also did not show any significant effect of harmine on memory (Abelaira et al. 2013). In our study, the effect of the stressor seemed to be transient, and on the day of the memory test, the effect of the social stressor already had resolved. Further studies are needed with stressors that are able to induce long-term cognitive impairment to assess the effect of harmine administration on stress-induced cognitive impairment.

Reduced levels of BDNF protein in specific regions of the brain has been associated with cognitive impairment (Knable et al. 2004; Saruta et al. 2010; Autry and Monteggia 2012; Reinhart et al. 2015; Chen et al. 2017). Treatment of brain disorders is generally accompanied by an alteration – usually an increase – in BDNF levels (Coppell et al. 2003; Lee and Kim 2008; Cooke et al. 2009). In this study, there was no effect of RSD or treatment with harmine on BDNF concentration in the frontal cortex or hippocampus. The absence of an effect of RSD on BDNF levels might be explained by the transient effect of the stressor used in this study, resulting in a normalization of BDNF levels at the time of assessment. Other studies suggest that assessment at an earlier time point could have shown changes in BDNF concentration (Hoffman et al. 2015). However, earlier assessment may potentially have been too soon to observe any effect of harmine treatment, as treatment with antidepressant drugs often takes at least 1 week to induce behavioral changes. Another point to take into consideration is the high variability in the BDNF concentration. Since normal variability in BDNF is high, increasing the sample size will increase the statistical power and may help to understand the effects of both RSD stress and harmine in BDNF concentration in future studies.

[^11^C]PBR28 PET imaging did not show any significant effect of the social stress protocol or harmine treatment on glial activation. It is known that microglial activation starts hours after exposure to the stressor and can last for several days or even weeks, decreasing gradually as the resolution of neuroinflammation begins (Schwartz and Baruch 2014). Kopschina Feltes and colleagues found a significant effect of the RSD protocol on [^11^C]PK11195 uptake 6 days after RSD in several key regions associated with depressive behavior (e.g., medial prefrontal cortex, entorhinal cortex, and insular cortex), but this effect was not observed anymore 3 weeks after RSD (Kopschina Feltes et al. 2019). In our study, apparently either the neuroinflammatory process was not severe enough to be shown by PET imaging or the neuroinflammatory response had already resolved 11 days after the last RSD trial. The latter option is in line with the results of the behavioral studies, which also did not elicit long-term changes in behavioral parameters associated with depressive-like behavior. [^11^C]PBR28 PET also did not show any significant effect of harmine treatment on tracer uptake, neither in controls nor in defeated animals. The latter is most likely due to the lack of an effect of RSD on glial activation at the time of the PET scan.

## Conclusion

Harmine was not able to reverse the RSD-induced acute anxiety and depressive-like behavior observed in the OFT and SPT shortly after exposure to the stressor. However, harmine treatment caused a number of side effects in control and RSD animals. Harmine treatment caused significant bodyweight loss, especially in animals exposed to the stress of the RSD protocol. In addition, harmine caused acute and delayed changes the locomotor behavior of animals, irrespective of the stressor. On the other hand, harmine did not alter the glial activation state of the brain or BDNF concentration in frontal cortex or hippocampus, regions that are key for stress regulation and further brain homeostasis. To better assess the antidepressant and anti-inflammatory effects of harmine, further studies using different stressors or longer-lasting RSD protocols to induce a chronic stress response in the organism are needed. In this context, it might be of interest to compare the effects of harmine in animals submitted to either RSD or CMS, as studies with these models appear to show different results.
